# The ZrO_2_ Formation in ZrB_2_/SiC Composite Irradiated by Laser

**DOI:** 10.3390/ma8125475

**Published:** 2015-12-14

**Authors:** Ling Liu, Zhuang Ma, Zhenyu Yan, Shizhen Zhu, Lihong Gao

**Affiliations:** 1 School of Materials Science and Engineering, Beijing Institute of Technology, Beijing 100081, China; hstrong929@bit.edu.cn (Z.M.); yanzhenyu@bit.edu.cn (Z.Y.); zhusz@bit.edu.cn (S.Z.); gaolihong@bit.edu.cn (L.G.); 2 National Key Laboratory of Science and Technology on Materials under Shock and Impact, Beijing 100081, China

**Keywords:** ZrB_2_/SiC, laser irradiation, ZrO_2_ formation

## Abstract

In order to clearly understand the details of ZrO_2_ formation during ablation, high intensity continuous laser was chosen to irradiate ZrB_2_/SiC. The results reveal that there are two different modes of ZrO_2_ formation depending on whether liquid SiO_2_ is present. When liquid SiO_2_ is present, ZrO_2_ generated by the oxidation of ZrB_2_ is firstly dissolved into SiO_2_. Then, ZrO_2_ will precipitate again, the temperature will decrease and the SiO_2_ will evaporate. Otherwise, the ZrB_2_ will be oxidized to ZrO_2_ directly.

## 1. Introduction

Ultra high temperature ceramics (UHTCs) refer to a class of refractory materials with melting temperatures in excess of 3000 °C, such as diborides and carbides of transition metals [1,2]. As a member of UHTCs, zirconium diboride (ZrB_2_) has been widely attractive for decades. Because of its excellent properties, such as high melting temperature, high thermal conduction, excellent mechanical properties, *etc.*, ZrB_2_ can be operated as leading edges in hypersonic vehicles.

According to previous research, ZrB_2_/SiC exhibited excellent ablation resistance against oxyacetylene torch, arc jet or plasma arc [3,4,5]. No obvious mass loss or macroscopic damage appears. Some products, like SiO_2_ and ZrO_2_, can be detected ona microscopic scale, and have been proved to be helpful to improve ZrB_2_/SiC’s ablation resistance [6,7]. However, the upper surface of the sample is wholly heated by the above ablation method, and we could only obtain the information of phase and microstructure until the ablated sample cools completely. Longer cooling time contributes to ablation transformation. So some important characterized information has been concealed or disappeared during the cooling process. Researchers only observed that the ablated layer of ZrB_2_/SiC was composed by ZrO_2_ skeleton and liquid SiO_2_ [8,9]. The real process and details of the formation of oxidation product, especially ZrO_2_, are still unknown.

If the information of phase and microstructure under high temperature can be preserved by fast cooling, the real transformation will be easily observed at room temperature. Because of thistheory, the high intensity laser ablation method with rapid heating rate was utilized. The local temperature of ZrB_2_/SiC at the spot center can reach thousands of degrees instantaneously at the beginning of laser irradiating. When laser loading stops, the sample can cool down rapidly to room temperature (which is similar to quenching), since the heating area is very small and the emissivity and conductivity of ZrB_2_/SiC are very high [10,11].

In this paper, ZrB_2_/20 vol % SiC composite (donated as ZS hereafter) was prepared and irradiated by high intensity continuous laser. The phase and microstructure evolution of ZrB_2_/SiC was investigated.

## 2. Results and Discussion

**Figure 1:**
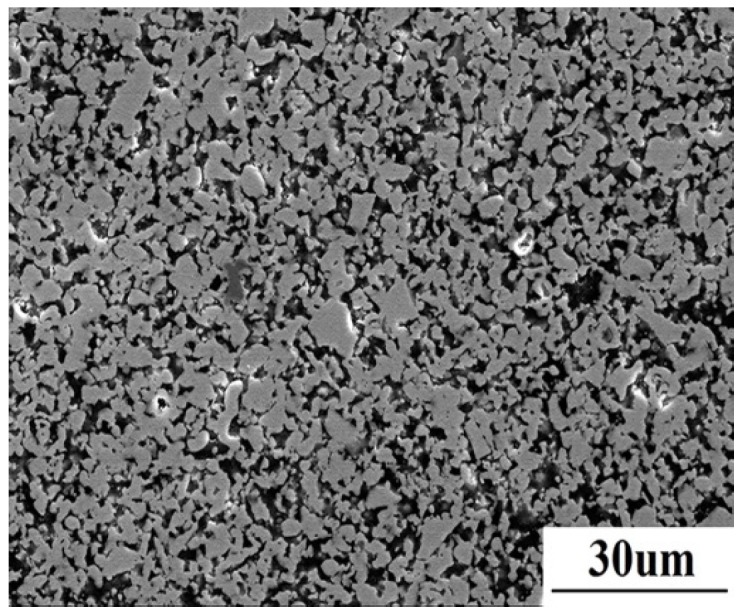
The microstructure of as-sintered ZrB_2_/SiC composite.

Figure 1 shows the polished surface microstructure of the as-sintered material. ZrB_2_ with grain size of about 5 μm is present as a grey matrix, while the dark SiC homogeneously distributes in the matrix.

**Figure 2:**
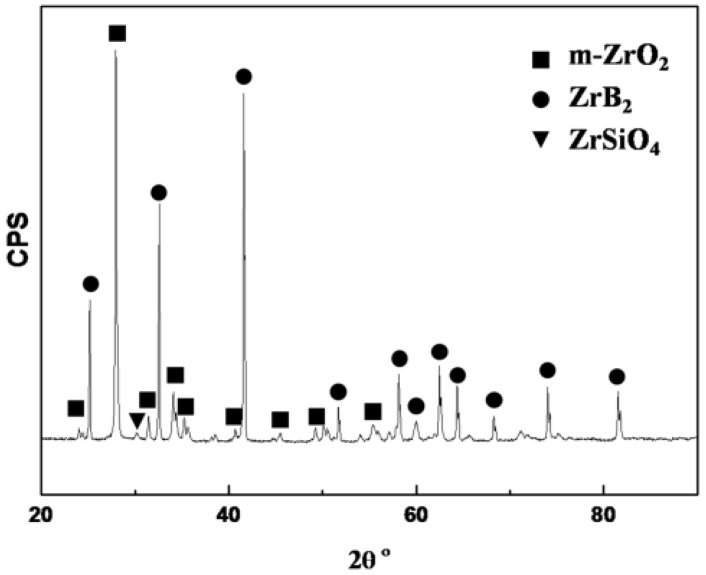
X-ray diffraction (XRD) of the sample surface afterablation for 20 s.

The X-ray diffraction (XRD) result of the ZrB_2_/SiC surface after laser ablation for 20 s is shown in Figure 2. According to the XRD, m-ZrO_2_ and ZrB_2_ are the major phases on the surface. It reveals that some ZrB_2_ are oxidized into ZrO_2_ while parts of ZrB_2_ are not oxidized at all. No peaks of SiO_2_ can be observed on the spectrum, because SiO_2_ is amorphous on the surface. However, small amounts of ZrSiO_4_ aredetected, as shown in the spectrum. This proves that SiO_2_ forms, and that parts of ZrO_2_ dissolve into SiO_2_ to form ZrSiO_4_.

**Figure 3:**
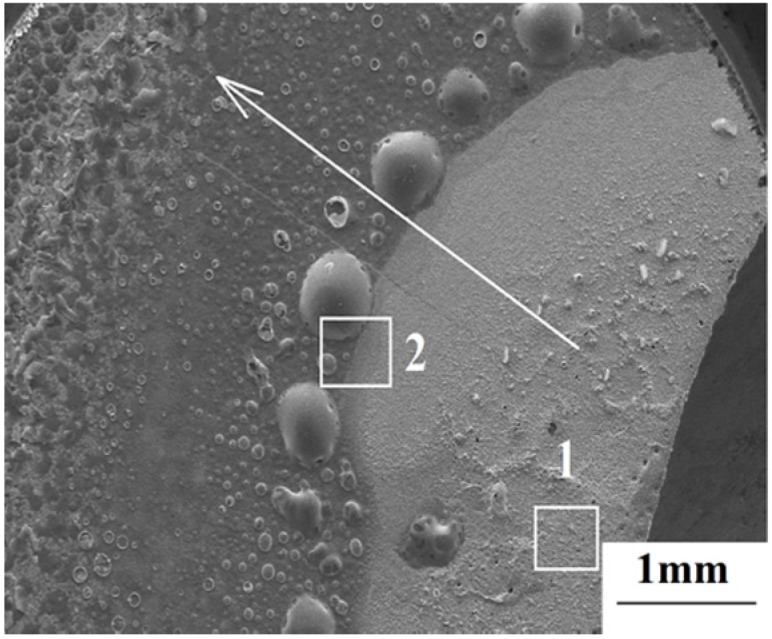
The surface macrostructure of the sample after ablation for 20 s.

The surface macrostructure of ZrB_2_/SiC after laser ablation for 20 s is shown in Figure 3. Since the local heating of the laser induces extremely high temperature at the spot center, a great temperature gradient is generated along the radial direction, shown bythe arrow in Figure 3. Significant differences in ablation behavior between area 1 and area 2 were detected.

**Figure 4:**
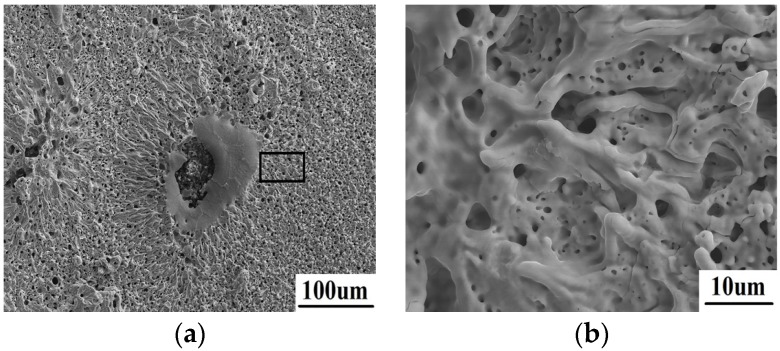
The microscopic morphology of area 1 (**a**) with a typical flushing morphology (**b**).

The surface morphology of area 1 after laser ablation is shown in Figure 4. From Figure 4a, the surface performs a porous structure. The energy dispersive spectroscopy (EDS) result reveals that only ZrO_2_ exists on the surface; neither ZrB_2_ nor SiC can be detected. A typical flushing morphology is clearly shown as Figure 4b. It should be attributed to the liquid splashing and the following rapid solidification. This means that the oxidized ZrO_2_ in this region has been totally melted during laser ablation. Meanwhile, SiC is totally decomposed because the temperature here is higher than its decomposition point of 2300 °C.

**Figure 5:**
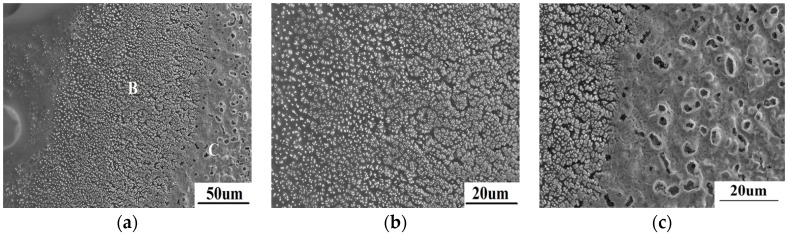
The microscopic morphology of area 2 (**a**) and the further amplified morphology of region B (**b**) and C (**c**).

The different microstructure in area 2 is shown in Figure 5. According to Figure 5a, lots of white or dark grey particles implant in amorphous substance. Based on the EDS result as shown in Table 1, the particles are ZrO_2_ while the amorphous substance is SiO_2_. This also can be confirmed by the XRD result shown in Figure 2. The region shown in Figure 5b is farther fromthe spot center than the region shown in Figure 5c. As can be seen in Figure 5b, very fine sub-micron ZrO_2_ distributes evenly in amorphous SiO_2_. With the temperature increases from left to right in Figure 5b, amorphous SiO_2_ reduces gradually, while the quantity and size of ZrO_2_ particles increase gradually. In Figure 5c, amorphous SiO_2_ is much lower, even almost disappearing at the right side, which results from the evaporation of SiO_2_. Moreover, ZrO_2_ grains close to the spot center grow gradually, and can even be seen to sinter obviously.

**Table 1:** The energy dispersive spectroscopy (EDS) results of area B and C in Figure 2.

Elements	Area B		Area C	
	Weight Ratio %	Atomic Ratio %	Weight Ratio %	Atomic Ratio %
O K	36.32	67.85	26.52	66.30
Si K	15.32	16.31	1.51	2.15
Zr L	48.36	15.85	71.97	31.55

**Figure 6:**
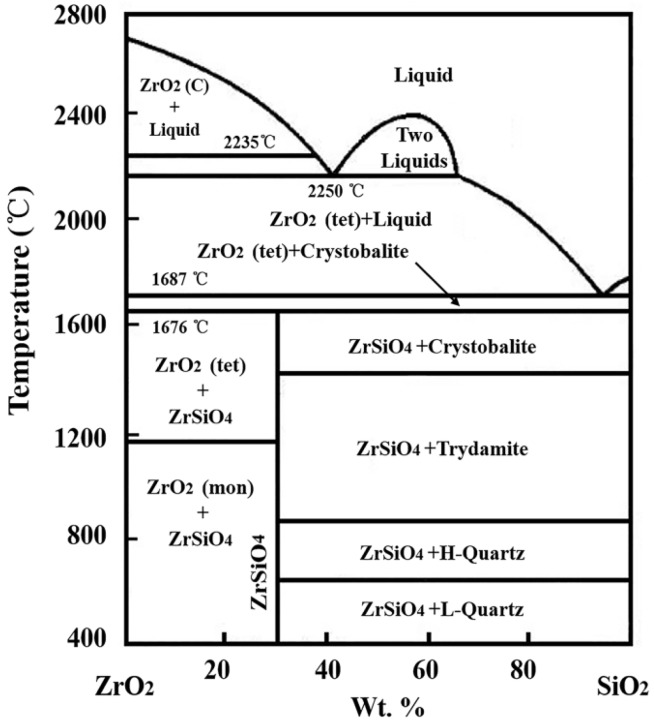
Thermal equilibrium phase diagram of SiO_2_-ZrO_2_.

According to the equilibrium phase diagram of ZrO_2_-SiO_2_ shown as Figure 6 [12], the melting temperature (2700 °C) of ZrO_2_ can be decreased by liquid SiO_2_, while ZrO_2_ has high solubility, about 43.3% in SiO_2_ at 2235 °C. With increasing temperature, the solubility of ZrO_2_ will increase gradually. When laser begins to irradiate on the material, the surface temperature soars up rapidly. A large number of ZrB_2_ are oxidized into ZrO_2_ between 600–700 °C. Meanwhile, SiC transforms into SiO_2_ at 1200 °C, and SiO_2_ exhibits as a liquid above 1400 °C. When the temperature approaches 1687 °C, the oxidized ZrO_2_ begins to dissolve into SiO_2_. SiO_2_ is unstable when the temperature is higher than 1800 °C [13]. The evaporation of SiO_2_ at high temperature makes the ZrO_2_ solubility decrease, so the extremely fine ZrO_2_ grains precipitate. With the evaporation proceeding, the solubility of ZrO_2_ dissolved into SiO_2_ significantly decreases. A lot of ZrO_2_ will precipitate from liquid SiO_2_ to form the morphology shown in Figure 5b. As precipitated ZrO_2_ is still subject to intense high temperature, fine ZrO_2_ grains with strong activity gradually grow up and sinter together for a short time to form the morphology as shown in Figure 5c. Therefore, the processing of ZrO_2_ dissolution and precipitation is the main transformation mechanism during ablation when liquid SiO_2_ is present.

**Figure 7:**
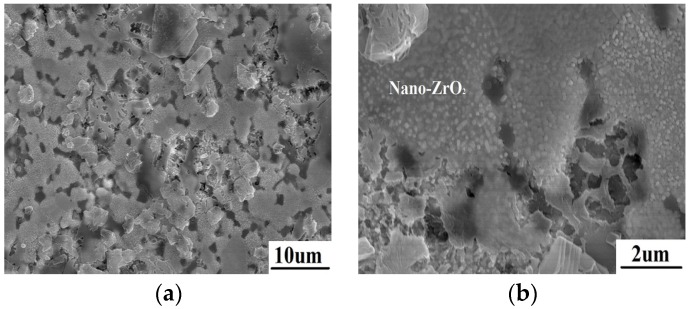
The morphology of ZrB_2_/SiC at the edge after ablation (**a**) with nano ZrO_2_ crystals packing on the surface (**b**).

The surface microstructure of ZrB_2_/SiC at the edge is shown in Figure 7. The temperature is lower than 1200 °C in this region away from the spot center. SiC oxidation and liquid SiO_2_ are not observed on the surface. The morphology of ZrB_2_ has changed because of oxidation. A lot of nano-crystals closely pack on the surface. This is proved to be ZrO_2_ according to the EDS result. This indicates that ZrB_2_ is directly oxidized into nano-ZrO_2_. The results reveal that there is another formation mechanism of ZrO_2_ during ablation when liquid SiO_2_ is absent.

## 3. Experimental Section 

The ZS composite was prepared using commercial powders listed as follows: ZrB_2_ (Alfa Aesar Ward Hill, MA, USA, 99.0%, particlesize ~50 μm); SiC (Alfa Aesar, 99.5%, particlesize 1–2 μm). The raw powders were accurately weighted by volume ratioof 4:1 and milled for 6 h in ethanol using zirconia milling media. The mixture powders were sintered by spark plasma sintering technology (SPS, DR. SINTER type 3.20, Fuji Electronic Industrial Co. Ltd., Kanagawa, Japan) at 1750 °C for 5 min with a rate of 200 °C/min. An axial pressure of 50 MPa was applied during the whole process.

The laser irradiation experiment in this paper was carried out by using ytterbium laser system (YLS-2000) (IPG Photonics Co. Ltd., Pittsfiels, MA, USA), with 1070 nm wave-length in atmospheric environment. The spot size of the Gaussianlaser was set as about 10 mm. The power density at the spot center reached 20 MW/m^2^ in Gaussian laser, and the duration time was 20 s. The dimensions of the ablation specimen were Φ25 mm × 3 mm.

The phase of the sample surface after ablation was detected by X-ray diffraction (XRD, X’pert PRO MPD, PANalytical B.V. Co. Ltd., Amsterdam, The Netherlands, Cu Kα). The surface microstructures of the sample before and afterablation were examined by scanning electron microscope (SEM, Philips S-4800, Hitachi Ltd., Yokohama, Japan). The composition of the sample was identified by energy dispersive spectroscopy (EDS, Oxford Instruments Co. Ltd., Oxfordshire, UK).

## 4. Conclusions

The ZrO_2_ formation in ZrB_2_/SiC composite during ablation was investigated in this paper. A characterized phenomenon at elevated temperature is preserved by using high intensity continuous laser with the rapid heating rate to irradiate. Two mechanisms of the ZrO_2_ formation are directly obtained, depending on whether liquid SiO_2_ is present. When liquid SiO_2_ is present, oxidized ZrO_2_ firstly dissolves into SiO_2_. Fine ZrO_2_ grains precipitate with the evaporation of liquid SiO_2_, and then grow, and even sinter together. Otherwise, the ZrB_2_ will be oxidized into ZrO_2_ directly.
